# The therapeutic effect of 5-nitro-8-hydroxyquinoline on *Aspergillus fumigatus*: results from *in vitro* and *in vivo* models of invasive aspergillosis

**DOI:** 10.3389/fcimb.2026.1843404

**Published:** 2026-05-26

**Authors:** Jiyu Ran, Xue Zhou, Zhongyan Gao, Xuanrong Huan, Meng Zeng, Zeng Tu

**Affiliations:** 1 Department of Clinical Laboratory, The Affiliated Yongchuan Hospital of Chongqing Medical University, School of Basic Medical Sciences, Chongqing Medical University, Chongqing, China; 2 Tuberculosis Reference Laboratory, Chongqing Tuberculosis Control Institute, Chongqing, China

**Keywords:** *A. fumigatus* infection, antifungal drugs, aspergillus fumigatus, invasive aspergillosis, quinolinol

## Abstract

*Aspergillus fumigatus* is an important opportunistic pathogen. In the immunocompromised people, *A. fumigatus* infection can cause invasive aspergillosis, which is characterized by high incidence rate and high mortality. However, due to the limited availability of anti-fungal drugs and the continuous emergence of drug-resistant strains, the treatment of *A. fumigatus* infection is facing enormous challenges. 5-nitro-8-hydroxyquinoline (NQ) is an ancient antibiotic and pharmaceutical intermediate and fungicide, which is not only exhibits good antibacterial activity on against drug-resistant *Staphylococcus aureus*, *Candida* spp., but also has good biofilm eradication on activity against *Pseudomonas aeruginosa*, *Acinetobacter baumannii*. However, it is not known whether NQ has anti-*A. fumigatus* infection effects *in vivo*. This study evaluates the *in vitro* and *in vivo* activity of NQ against *A. fumigatus*. The results showed that NQ not only had a good anti-*A. fumigatus* effect, but also promoted the phagocytosis and killing ability of alveolar macrophages to *A. fumigatus*. In addition, we found that intraperitoneal injection of NQ significantly reduced the diffuse infection degree of *A. fumigatus* and the fungal burden in mice tissues, and increased the secretion of IL-2, IL-6, IL-10 in the blood. NQ exerted a good anti-fungal effect. In summary, these results confirm that NQ may be a potential anti-fungal drug for treating *A. fumigatus* infection.

## 1 Introduction

*Aspergillus fumigatus* is a common opportunistic pathogen that can directly produce conidia in the air and spread and be inhaled into the lungs. When the body’s immune system is weakened, it can cause various aspergillosis and even life-threatening infections (Gülmez et al., 2021). According to some reports, the number of patients worldwide infected with *A. fumigatus* each year exceeds 8 million (Agarwal et al., 2022; Bongomin et al., 2017). Common aspergillosis includes allergic bronchitis pulmonary aspergillosis, chronic pulmonary aspergillosis (Eraso et al., 2020), and invasive pulmonary aspergillosis (IPA) (Sehgal et al., 2020), with the most severe being invasive pulmonary aspergillosis (IA) (Snelders et al., 2011), which has a mortality rate of over 50% (Brown et al., 2012). In stem cell transplant patients, the incidence of IPA is 43%, and in solid organ transplant patients, the infection rate exceeds 50% (Latgé and Chamilos, 2019). The commonly drugs used to against *A. fumigatus* in clinical are triazole drugs, such as itraconazole (ITR), posaconazole (POS), and voriconazole (VRC). Among them, VRC is the preferred drug for treating *A. fumigatus* infection. But in recent years, the resistance of *A. fumigatus* to triazole drugs has been increasing, and the mortality rate of patients infected with azole-resistant *A. fumigatus* can reach up to 100% (Van der Linden et al., 2015; Meis et al., 2016).

5-nitro-8-hydroxyquinolinel (NQ) is an ancient antimicrobial, as well as an important pharmaceutical intermediate and fungicide, with a wide range of pharmacological applications and unique mechanisms of action (Repac Antić et al., 2022). Due to the good safety of NQ, it has been used as a substitute for furantoin in the treatment of urinary tract infections in clinical practice (Kranz et al., 2018). NQ not only has good therapeutic effect on urinary tract infection, but also on bladder cancer, but also has good antibacterial activity against drug-resistant *Staphylococcus aureus*, yeast, and good biofilm eradication activity against *Pseudomonas aeruginosa*, *Acinetobacter baumannii* (Sobke et al., 2012; Abouelhassan et al., 2017; Fuchs et al., 2021). The European Union Clinical Sensitivity Classification System (EUCAST) sets a clinical threshold for the minimum inhibitory concentration (MIC) of *Escherichia coli* in urinary tract infections, which is 16 mg/L, but the threshold for other pathogens has not been determined yet. It is worth noting that the drug also exhibits activity *in vitro* against microorganisms with therapeutic challenges, such as drug-resistant *Mycobacteria* (Hoffmann et al., 2023), including *Mycobacterium tuberculosis* (Hoffmann et al., 2022).

In the face of the constantly emerging new drug-resistant *A. fumigatus* and the limited availability of anti-fungal drugs, doctors often feel helpless when patients with *A. fumigatus* infections. Therefore, the development of new drugs to against *A. fumigatus* is urgent. This study confirmed through an IA mouse model that NQ has a good therapeutic effect on *A. fumigatus* infection, providing data for the development of new drugs against *A. fumigatus* infection, and offering new strategies for clinical treatment of *A. fumigatus* infection.

## 2 Materials and methods

### 2.1 Strains and reagents

The test strain include the standard strain of *A. fumigatus* (AF293) and other clinically isolated *A. fumigatus* strains AF1 and AF2 (ITR resistant strains), AF4 and AF70 (VRC resistant strains). Before the experiment, the *A. fumigatus* strain was inoculated onto potato agar (PDA) plates and cultured at 37 °C for 3–5 days, followed by two consecutive passages. Mouse alveolar macrophages (MH-S cells) were cultured in complete Dulbecco’s modified Eagle’s medium (Thermo Fisher Scientific, Waltham, MA, USA) containing 10% fetal bovine serum (Jiangsu MRC Biotechnology Co., Ltd., Jiangsu, China) and 1% penicillin streptomycin (100 ×) (McLean Biochemical Technology Co., Ltd., Shanghai, China) at 37 °C and 5% carbon dioxide. NQ (purity > 98%) was purchased from McLean Biochemical Technology Co., Ltd. (Shanghai, China). ITR and NQ were dissolved in Dimethyl sulfoxide to a concentration of 5.12 mg/mL, and stored at -20 °C for future use.

### 2.2 The drug sensitivity of NQ to *A. fumigatus*

The drug sensitivity of NQ to AF293 was determined by the microliquid-based dilution method of M38-A2 filamentous fungi of the Clinical Laboratory Standards Institute (CLSI) of the United States, and the MIC_90_ value was obtained (Espinel-Ingroff et al., 2010; Cenci et al., 2000). *A. fumigatus* conidia were resuspended in RPMI-1640 medium (Thermo Fisher Scientific, Waltham, MA, USA) at a concentration of 1.0×10^5 cells/mL. Then, the conidia suspension was co-cultured with NQ at 37 °C for 48 h by serial dilution method, and the absorbance of the multifunctional enzyme marker (Thermo Fisher Scientific, Waltham, Massachusetts, USA) at 600 nm was measured. The concentration range of the drugs was: ITR from 0.25 to 32 μg/mL, and NQ from 0.25 to 1024 μg/mL. Each group had three replicates, and the mean value was taken. The experiment was independently repeated three times.

### 2.3 Cell activity test

The effect of NQ on the viability of MH-S cells was measured by the Cell Counting Kit-8 (CCK-8) assay kit (Apexbio Technology LLC, Houston, Texas, USA) (Wang et al., 2022). In brief, MH-S cells were seeded at a density of 2×10^5 cells/mL into 96-well plates and cultured overnight. Then, the cells were further cultured for 4 h in different concentrations of NQ ranging from 0 to 60 μg/mL. Subsequently, 10 μL of CCK-8 reagent was added to each well and the plates were incubated at 37 °C for another 2 h. The absorbance of the multifunctional enzyme marker at 490 nm was measured. Each experiment was independently repeated three times.

### 2.4 Phagocytosis and killing assay

Firstly, the MH-S cells was cultured to a density of 80%-90%, resuspend the cells, and prepare a cell suspension with a concentration of 1×10^5 cells/mL. Then, inoculate 1 mL of the cells suspension onto a 12-well plate with cell slides, and incubate overnight under the conditions of 37 °C and 5% CO_2_. Subsequently, prepare the NQ solution and the *A. fumigatus* conidia suspension (1×10^6 cells/mL) for later use. Removed the 12-well plate from the incubator, add the NQ solution (working concentrations is 0, 5, 10, 20 μg/mL) to co-culture with the MH-S cells for 4 h, discard the supernatant, and then add 1 mL of the prepared conidia suspension to co-culture with the MH-S cells for 2 h. Next, add pre-cooled (4 °C) PBS solution to stop the phagocytosis and wash away the unphagocytosed conidia, then discard the PBS in the well, and add 5 μg/mL calcium fluorescence white solution, stain for 2–3 minutes, and then wash 2–3 times with PBS. Finally, take the cell slides out, then observe and photograph them under a microscope, randomly count 200 cells. The experiment was independently repeated three times. The calculation formula is as follows: Conidia phagocytosis rate = Cells phagocytosing conidia/200 × 100%.

The previous part was consistent with the phagocytosis test culture. Pre-cooled PBS was added to wash away the non-phagocytosed conidia, then 1 mL of sterilized water was added to lyse the cells, the suspension was collected, 100 μL of the suspension was mixed and evenly spread on a PDA medium plate, and cultured at 37 °C for 36 h before counting the colonies.

### 2.5 The *in vivo* therapeutic effect of NQ to IA

#### 2.5.1 The drug safety verification of NQ

Female mice, C57 (n = 24; age 6–8 weeks; weight 20–25 g; Southwest Medical University Animal Experiment Center, Luzhou, Sichuan) were randomly divided into 4 groups: the untreated negative control group (blank group), the group injected with normal saline only (control group), the ITR (75/mg/kg/d) group, and the NQ (80mg/kg/d) group (Feng et al., 2021; Shim et al., 2010), except for the blank group, the control group was injected with an equal amount of normal saline as the group of NQ (80mg/kg/d) every day for 5 days. The weight of the mice was recorded. On the 5th day, the mice were euthanized, and their blood was collected for liver and kidney function tests. The liver and kidney tissues of the mice were also taken for Hematoxylin- Eosin (HE) staining to observe whether there were any pathological changes.

#### 2.5.2 The therapeutic effect of NQ on IA

Female mice, C57 (n = 70; 6–8 weeks; 20–25 g; Southwest Medical University Animal Experiment Center, Luzhou, Sichuan) were randomly divided into 7 groups: the untreated negative control group (blank group), the group injected with normal saline only (control group), the model group (only infected with *A. fumigatus* conidia without drug treatment), ITR (75/mg/kg/d), NQ (20 mg/kg/d), NQ (40 mg/kg/d), and NQ (80 mg/kg/d). Except for the blank group and the control group, the mice in the other groups were intravenously injected with *A. fumigatus* conidia suspension of 50 μL (10×10^7 cells/mL) through the tail vein (Zhang et al., 2022). 2 h after the IA mouse model was successfully established, the treatment was administered by intraperitoneal injection.

The therapeutic efficacy of NQ in treating IA mice was evaluated by monitoring the fungal burden and conducting histopathological assessment. After 5 days of treatment, samples of liver, kidney, lung and blood were collected from each group of mice. Half of each of the collected liver, kidney and lung samples were selected for preparing the dilution solution for making the homogenate, and were cultured on PDA medium at 37 °C for 36 h, and the number of colony-forming units per gram of tissue was determined. The remaining tissues were fixed with 10% methanol and embedded in paraffin. Thin sections were cut and stained with Periodic Acid-Schiff (PAS) and HE for microscopic observation. The blood of mice was used to detect cytokine.

### 2.6 Data analysis

Each experiment was independently conducted three times for statistical analysis. GraphPad Prism v9.0 (GraphPad Software, Inc., San Diego, CA, USA) was used to draw the charts. Student’s *t*-test or one-way ANOVA was employed for comparisons between groups. *P* < 0.05 was considered statistically significant.

## 3 Results

### 3.1 NQ exhibits anti-fungal activity to *A. fumigatus*

The MIC_90_ of NQ for the 5 test strains is 16 μg/mL (**Table 1**). We selected the *A. fumigatus* standard strain (AF293) for the subsequent research.

**Table 1:** Anti-fungal activity of NQ against *A. fumigatus*.

Strain	FLC(μg/mL)	ITR(μg/mL)	VRC(μg/mL)	AmB(μg/mL)	NQ(μg/mL)
AF293	>64	0.25	0.13	0.25	16
AF1	>64	>32	0.25	0.25	16
AF2	>64	>32	0.5	<0.25	16
AF4	>64	1	32	0.5	16
AF70	>64	1	32	<0.25	16

AF293 is the standard strain of *A. fumigatus*, AF1 and AF2 are ITR resistant strains, AF4 and AF70 are VRC resistant strains, the MIC_90_ of NQ for all strains is 16 μg/mL. `AmB, amphotericin B; FLC, fluconazole; ITR, itraconazole; VRC, voriconazole; NQ, 5-nitro-8-hydroxyquinoline.

### 3.2 NQ enhances the ability of MH-S cells to phagocytose and kill *A. fumigatus* conidia

**Figure 1:**
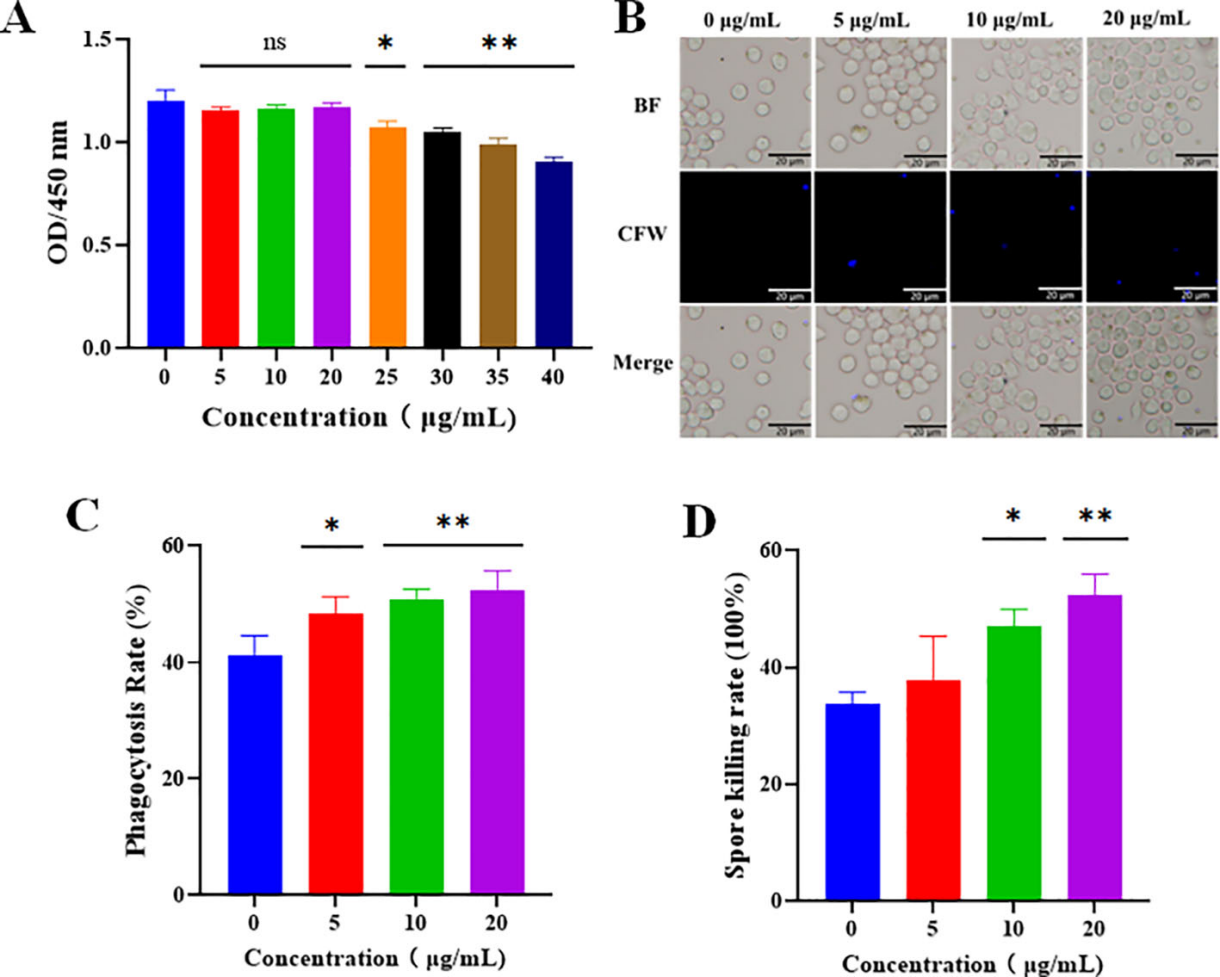
NQ promotes the phagocytic and killing ability of MH-S cells towards *A. fumigatus* conidia. **(A)** The vitality of MH-S cells after co-culture with NQ for 4 h. **(B, C)** MH-S cells were cultured with NQ for 4 h, followed by the addition of *A. fumigatus* conidia at an MOI of 5, then MH-S cells was observed with a fluorescence microscope, scale bar = 20 μm. BF, bright field; CFW, calcium fluorescent white stain. **(D)** and the CFU was calculated to evaluate the killing ability of MH-S cells. ns = not significant. *p < 0.05 and **p < 0.01 *vs*. MH-S cells treated with NQ at 0 μg/mL.

After co-culturing 20 μg/mL NQ with MH-S cells for 4 h, the survival rate of MH-S cells exceeded 90% (**Figure 1A**). To determine whether NQ affects the phagocytic ability of MH-S cells, MH-S cells were incubated with NQ for 4 h and then co-cultured with *A. fumigatus* conidia for 2 h. The results showed that the phagocytic ability of MH-S cells for conidia significantly increased (**Figures 1B,C**). Additionally, the effect of SNH on the ability of MH-S cells to kill conidia of *A. fumigatus* was determined by calculating the number of CFU. The results indicated that NQ significantly enhanced the killing ability of MH-S cells against *A. fumigatus* conidia (**Figure 1D**), and NQ increased the phagocytic and killing ability of MH-S cells against *A. fumigatus* conidia in a concentration-dependent manner.

### 3.3 NQ has a significant therapeutic effect on IA

**Figure 2:**
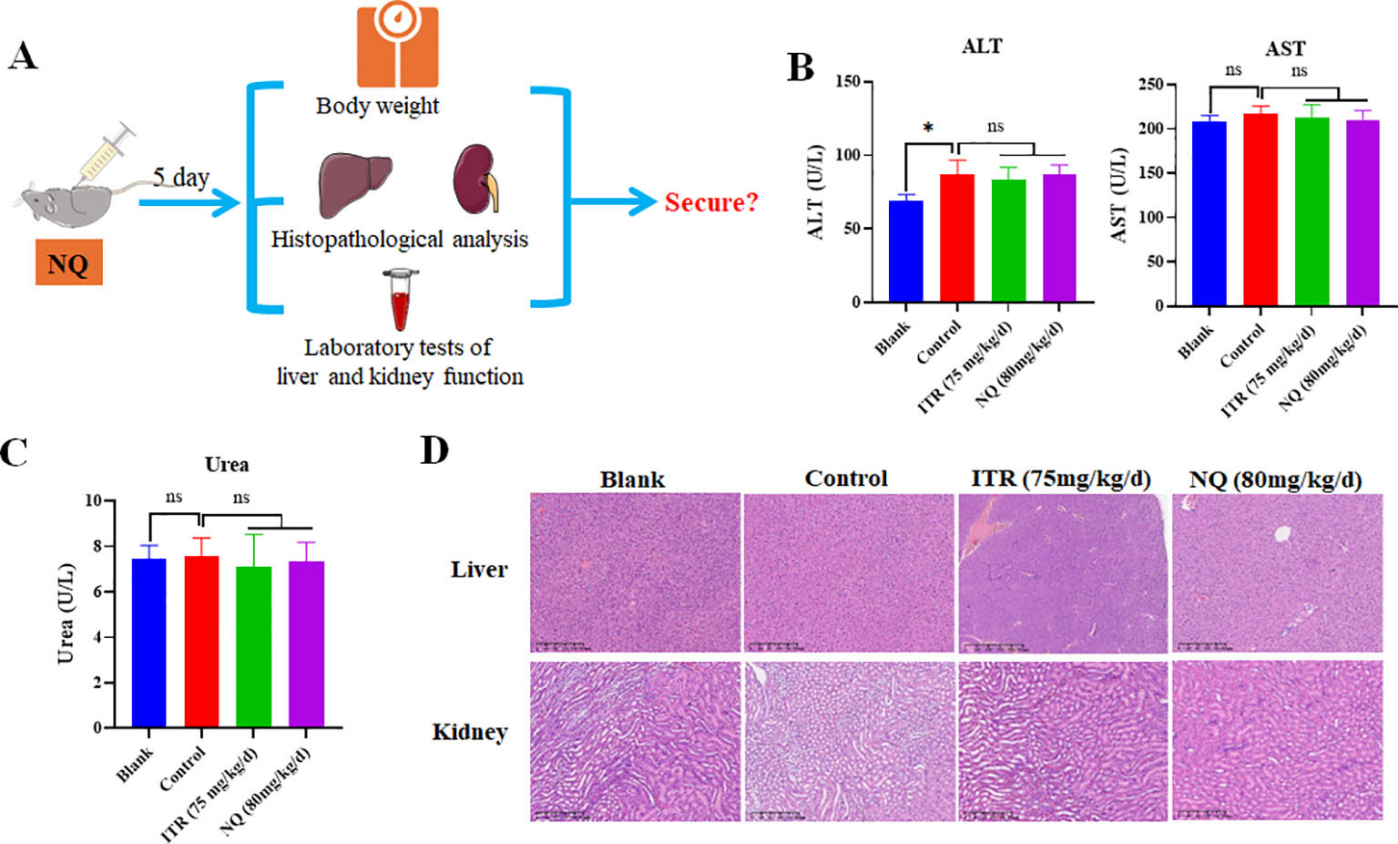
Safety verification of intraperitoneal injection of NQ in mice. **(A)** Schematic diagram of experimental model. After continuous intraperitoneal injection of NQ (80 mg/kg/d) for 5 days, the levels of ALT, AST, and urea in the blood of mice **(B, C)**, as well as the HE staining results of liver and kidney tissues **(D)**.

The *in vivo* safety of NQ was evaluated by intraperitoneal injection model, and blood and liver/kidney tissues of mice were collected for analysis (**Figure 2A**). The blood test results showed that there were no differences in alanine transaminase (ALT), aspartate transaminase (AST) and Urea among the control group, the ITR (75 mg/kg/d), and NQ (80 mg/kg/d) drug injection groups (**Figures 2B, C**). Additionally, compared with the control group, there were no significant changes in liver and kidney pathological sections in the ITR (75 mg/kg/d) and NQ (80 mg/kg/d) drug injection groups (**Figure 2D**). Therefore, treatment with a concentration of 75 mg/kg/d of ITR and 80 mg/kg/d of NQ had no significant toxic side effects on mice.

**Figure 3:**
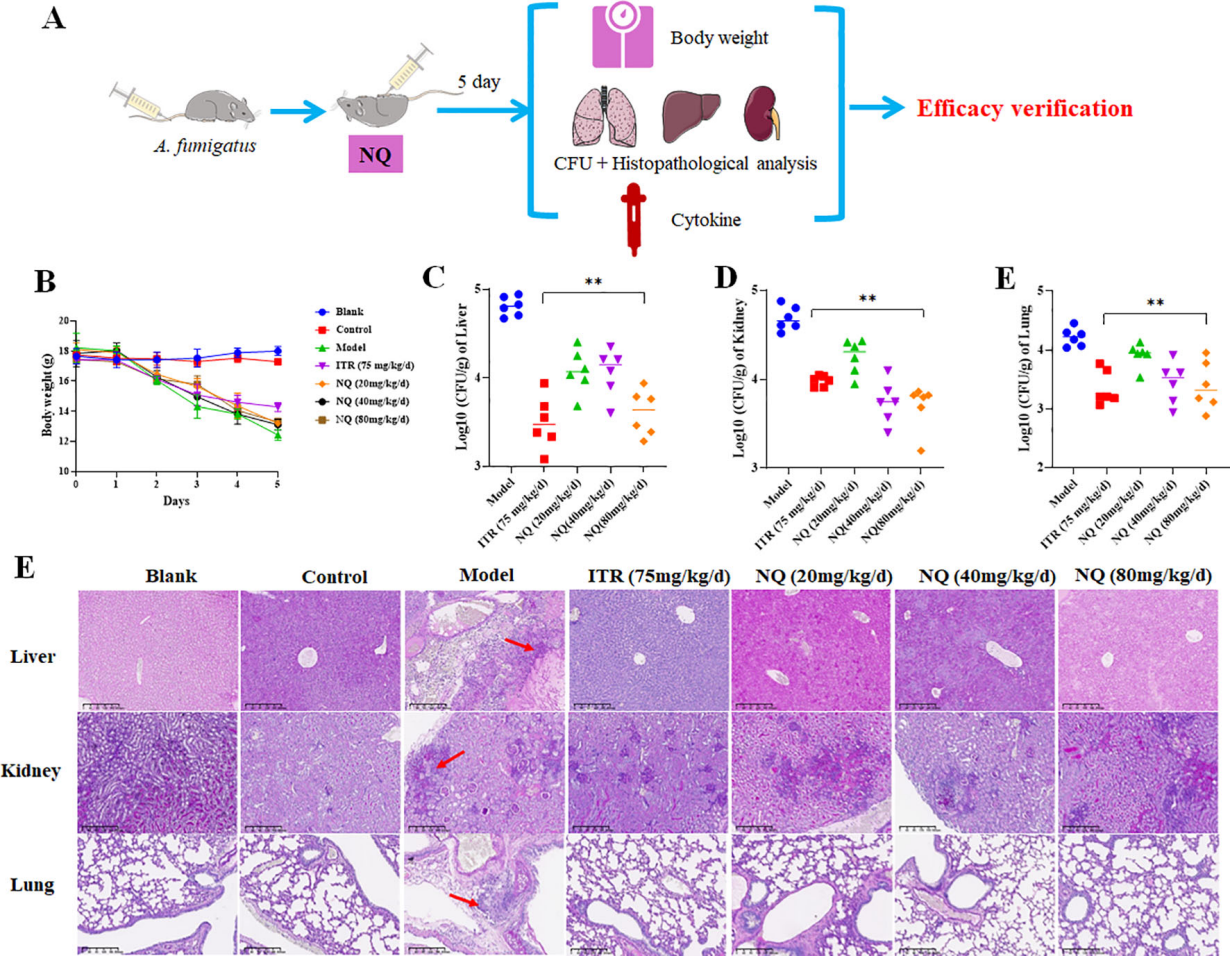
NQ alleviates tissue invasion of *A. fumigatus* conidia in IA mice. **(A)** Schematic diagram of experimental model. **(B)** After NQ treatment, the weight loss of IA mice slowed down. **(C-F)** Fungal burden in mouse liver, kidney, and lung tissues after 5 days of NQ treatment. Scale bar = 100 µm. *p < 0.05, **p < 0.01 *vs*. the model group.

**Figure 4:**
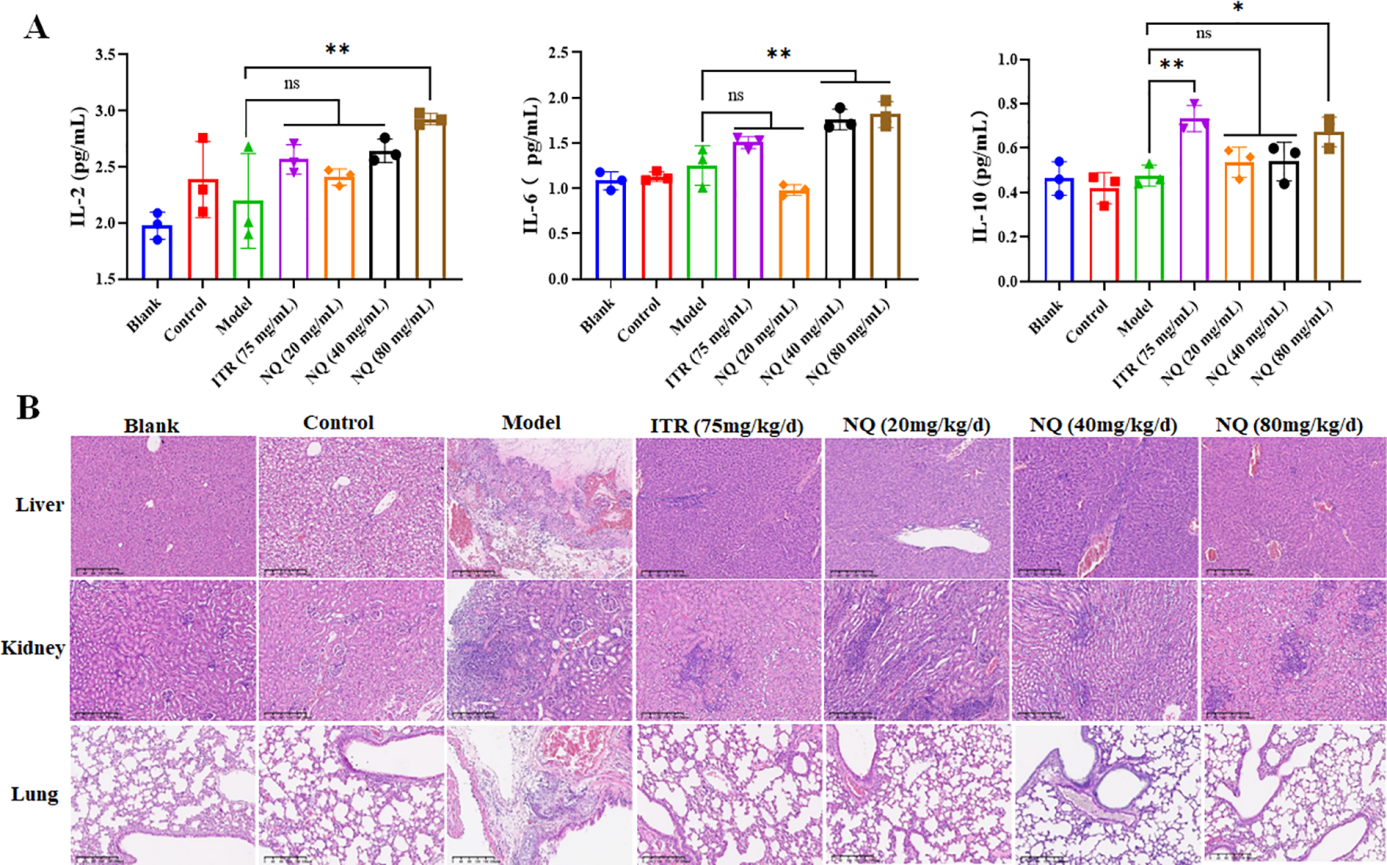
NQ alleviates tissue inflammation in IA mice. After treating IA mice with NQ for 5 days, it can regulate the secretion of IL-2, IL-6 and IL-10 in the blood of IA mice **(A)**. At the same time, HE staining observation shows that NQ treatment can reduce the inflammatory infiltration in liver, kidney and lung tissues **(B)**. Scale bar = 100 µm. *p < 0.05, **p < 0.01 *vs*. the model group.

The IA mouse model was used to evaluate the *in vivo* antifungal efficacy of NQ (**Figure 3A**). Compared with the model group, the weight of mice in the NQ treatment group decreased more gradually (**Figure 3B**), and the CFU counts in liver, kidney, and lung tissues were significantly reduced (**Figures 3C–E**). The PAS staining of pathological tissues of the mice showed that obvious filamentous fungal hyphae could be observed in the liver, kidney, and lung tissues of the model group (**Figure 3F**), which was consistent with the results of tissue fungal burden statistics. To evaluate the regulatory effect of NQ on inflammation in IA mice, we detected the levels of cytokines such as IL-2, IL-6, and IL-10 in the blood of mice. The results showed that compared with the model group, the levels of IL-2, IL-6, and IL-10 in the NQ (80 mg/kg/d) treatment group of mice were significantly increased (**Figure 4A**). Microscopic observation of the liver, kidney, and lung tissues of mice using HE staining showed that the degree of tissue inflammation was significantly reduced in the ITR and NQ treatment groups (**Figure 4B**).

## 4 Discussion

Among individuals with compromised immune systems, IA is the most destructive aspergillus-related disease (MaChado et al., 2024). Currently, due to the emergence of an increasing number of individuals with weakened immune systems and the rise in fungal drug resistance, not only is the spread of pathogenic fungi worldwide on the rise, but the incidence and mortality rates of fungal diseases are also increasing (Rudramurthy et al., 2019; Chen et al., 2024; Ikuta et al., 2024). What is particularly noteworthy is that in recent years, not only has the awareness of common invasive opportunistic fungal infections (such as *Candida albicans*, *Cryptococcus neoformans*, and *A. fumigatus*) continuously increasing, but also the number of patients with infections caused by emerging pathogenic fungi such as *Candida glabrata* and *Candida auris* has been on the rise (Staniszewska, 2020; Colombo et al., 2017). In this situation, the efficacy of anti-fungal drugs plays a decisive role. Unfortunately, the current anti-fungal drugs do not provide satisfactory treatment results, and the development of new anti-fungal drugs is also very slow. This not only increases the difficulty for clinicians in managing fungal infections, but also makes fungal infections become a more and more serious medical problem (Pathakumari et al., 2020; Zhang et al., 2021). Therefore, the development of new drugs with anti-fungal properties is urgently needed. Drug repurposing might be a promising strategy for developing new treatments for anti-fungal infections. Several non-antifungal drugs that have been reported, such as immunosuppressants and statins, have shown good anti-fungal infection effects (Zhang et al., 2021; Kim et al., 2020). Previous studies have confirmed that NQ has antibacterial effects on clinical isolates of *A. fumigatus* and *A. flavus* (Hoffmann et al., 2025). However, few studies have been conducted at the cellular and animal levels to explore whether it has therapeutic effects on *A. fumigatus* infection. This study is the first to confirm *in vivo* and *in vitro* that NQ not only promotes anti- *A. fumigatus* infection at the cellular level, but also demonstrates good therapeutic effects on systemic *A. fumigatus* infection at the animal level.

The conidia of *A. fumigatus* mainly enter the body through the respiratory tract and reach the lungs, thereby triggering a series of pulmonary inflammatory responses. It is well known that macrophages play a crucial role in the recognition, clearance, and inhibition of germination of *A. fumigatus* conidia (Rosowski et al., 2018; Espinosa and Rivera, 2016). In order to against the infection, the macrophages in the infected area of the host are activated, promoting the recruitment of neutrophils and T cells, which work together to resist the infection caused by *A. fumigatus* (Brown et al., 2012). In this study, we found that co-culturing macrophages with NQ significantly enhanced the phagocytic and killing capabilities of macrophages against *A. fumigatus* conidia (**Figure 1**). However, in immunocompromised individuals, the immune response in the lungs alone is not sufficient to completely eliminate the *A. fumigatus* conidia that enter the body. Therefore, some patients will develop systemic IA as their condition worsens.

This study established an IA model in mice to investigate whether NQ has an anti-*A. fumigatus* infection effect *in vivo*. Liver function biomarkers, ALT and AST, are key indicators for liver biosynthesis and metabolism (Liu et al., 2023), ALT and AST are usually included in routine blood tests and are widely used to assess liver health (Newsome et al., 2018; He et al., 2021). In the human body, Urea is the final product of nitrogen metabolism, when the concentration of Urea in the body is too high, it can lead to digestive disorders, renal dysfunction, and nephrotic syndrome, etc (Botewad et al., 2023). Therefore, ALT, AST and Urea have significant implications for predicting whether the host’s liver and kidney functions are normal. In this study, we found that compared to the control group, the NQ (80 mg/kg/d) administration group showed no significant changes in ALT, AST and Urea in the mice’ blood after continuous intraperitoneal injections for 5 days. At the same time, microscopic observation revealed that there were no significant pathological changes in the liver and kidneys of mice in each group (**Figure 2**). This suggests that the selected drug concentration is within the safe range.

Compared with the model group, after 5 days of NQ treatment, the fungal burden in the liver, kidney and lung tissues of the mice was significantly reduced (**Figures 3C–E**), and the infiltration of *A. fumigatus* hyphae in the interstitial spaces of each tissue was significantly decreased (**Figure 3F**). At the same time, the infiltration of inflammatory cells in each tissue among the drug treatment group was significantly reduced (**Figure 4B**), indicating that NQ is an effective therapeutic drug against *A. fumigatus* infection. In recent years, with the development of science and technology, more and more evidence has shown that the progression of human disease courses is closely related to the immune status the host. Studies have confirmed that the imbalance of Th cells and the secretion disorder of related cytokines are related to the pathogenesis of IA (Cenci et al., 1999). The activation of T cells is the most effective protective immune response in fungal infections (Lass-Flörl et al., 2013). The pro-inflammatory factors IL-2 and IL-6 play a protective role in fungal infections (Bellocchio et al., 2004). And the production of IL-2 is associated with the activation of Th1 cells and the stimulation of anti-fungal effector cells. IL-6 is involved in the regulation of the differentiation of Th2 and Th17 cells (Egwuagu, 2009). Previous studies have also confirmed that IL-6^-/-^ mice exhibit increased tissue inflammatory pathology, reduced phagocyte anti-fungal effect function, and impaired Th1 cell response after infection with *A. fumigatus* (Cenci et al., 2001). This indicates that IL-6 plays a protective role in the defense against *A. fumigatus* infection, IL-10 can regulate the activation of Th2 cells and the exertion of their anti-fungal effects, however, excessive levels of IL-10 will promote the invasion of fungal into tissues (Roilides et al., 2001; Cenci et al., 2000). In this study, by testing the blood of mice, it was found that NQ promoted the secretion of IL-2, IL-6 and IL-10 in mice in a concentration-dependent manner. Therefore, further research is needed to confirm the optimal drug treatment concentration and how cytokines function in the treatment of *A. fumigatus* infection.

This study aims to confirm the *in vitro* and *in vivo* anti-fungal activities of NQ against *A. fumigatus*. Our results indicate that NQ has an anti-*A. fumigatus* infection effect. The analysis data from the IA mice model in this study support this view, that is, NQ can exert therapeutic effects by regulating the ability of host immune cells and the secretion of cytokines. So NQ has the potential practicality in treating *A. fumigatus* infections.

## Data Availability

The original contributions presented in the study are included in the article/supplementary material. Further inquiries can be directed to the corresponding authors.
